# Multiple isolated spinal aneurysms – A rare condition with uncertain treatment strategies: A case report and literature review

**DOI:** 10.1016/j.bas.2025.104192

**Published:** 2025-01-02

**Authors:** F.M.C. Lioi, D. Dahlberg, J. Sundseth, K.B. Olsen, H.L. Lilleby, B. Nedregaard, M.K.H. Wiedmann

**Affiliations:** a Department of Neurosurgery, Oslo University Hospital, Oslo, Norway; b Department of Neurosurgery, Policlinico Umberto I, Sapienza University of Rome, Rome, Italy; c Department of Pathology, Oslo University Hospital, Oslo, Norway; d Department of Radiology and Nuclear Medicine, Oslo University Hospital, Oslo, Norway

**Keywords:** Spine, Aneurysm, Hematoma, Hemorrhage, Vascular malformation, Treatment

## Abstract

**Purpose:**

Isolated spinal aneurysms (iSAs) are rare, with an uncertain natural history and no established treatment guidelines. Multiple iSAs are even more uncommon, complicating treatment decisions.

**Methods:**

This study reports a case of a ruptured radiculo-pial artery aneurysm in a patient with multiple iSAs, treated with surgical excision, assisted by intraoperative neurophysiological monitoring (IONM). Further, we review and analyze all previously reported cases of multiple iSAs.

**Results:**

A 67-year-old woman with spinal subarachnoid hemorrhage and spinal cord compression due to a ruptured radiculo-pial artery aneurysm was treated surgically. Digital subtraction angiography (DSA) showed three spinal aneurysms. Intraoperative indocyanine green video-angiography (ICG-VA) revealed an aneurysm contributing to the left posterior spinal artery. The aneurysm was excised after proximal inflow occlusion under IONM. The other aneurysms spontaneously regressed, and the patient had a good functional outcome. Through a systematic literature review, we analyzed 13 multiple iSAs including our case, involving 34 aneurysms in total.

**Conclusions:**

There is no standardized treatment approach for multiple iSAs. These aneurysms are often fusiform and clustered in contiguous metameric regions. They can be classified into flow-related and wall-weakening aneurysms. Surgery offers definitive treatment for ruptured iSAs and relieves spinal cord compression. Due to the common fusiform shape, parent vessel sacrifice may be necessary, and should include IONM and ICG-VA to minimize complications. Conservative treatment is viable as spontaneous regression often occurs.

## Highlights

• Isolated spinal aneurysms (iSA) are a rare disease. Multiplicity of iSAs further complicates treatment decisions.
• There are several treatment options, including surgery, endovascular treatment or conservative management.
• Surgery offers definitive treatment for ruptured iSAs and relieves spinal cord compression.
• Some of the non treated aneurysms regress spontaneously over time.

## Abbreviation list:

DSA: digital subtraction angiography
ICG: indocyanine green
IONM: intraoperative neurological monitoring
IQR: interquartile range
iSA: isolated spinal aneurysm
MALT: Mucosa-associated lymphoid tissue
MRA: magnetic resonance angiography
MRI: magnetic resonance imaging
mRS: Modified Rankin Scale
SAH: subarachnoid hemorrhage

## 1 Introduction

Isolated spinal aneurysms (iSAs) are rare vascular anomalies that remain poorly understood and are distinct from secondary spinal aneurysms, associated with spinal vascular malformations. Although iSAs most often occur sporadically, several predisposing factors, such as bilateral vertebral stenosis, aortic coarctation, and connective tissue disorders, have been identified as potential contributors to their development (Alomari et al., 2022). The occurrence of multiple iSAs is exceedingly rare and presents significant challenges, particularly in devising effective management strategies. Treatment options may include surgical interventions, endovascular procedures or conservative management, since spontaneous resolution of those aneurysms has been reported (Zhou et al., 2024; McGuire et al., 2023; Limaye et al., 2021; Turrini et al., 2021; Alomari et al., 2022; Abdalkader et al., 2021; Ikeda et al., 2016). However, a standardized treatment algorithm to manage the complexities associated with multiple iSAs has yet to be established. In this manuscript we present a case of a patient with multiple iSAs who presented with hemorrhage and we conduct a comprehensive review and analysis of reported cases of multiple iSAs in the literature.

### 1.1 Case presentation

**Fig. 1:**
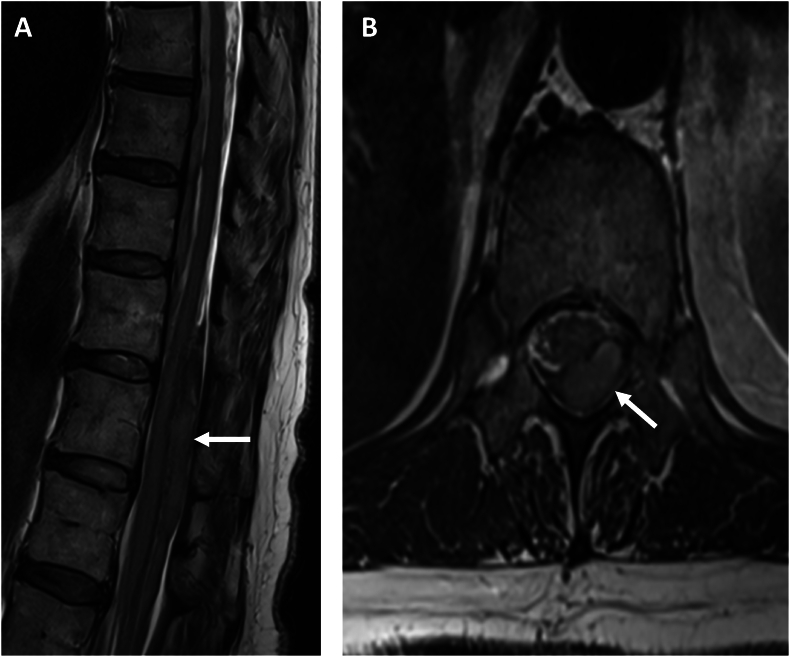
Sagittal (A) and axial (B) T2-weighted MRI showing an intradural spinal hematoma exerting mass effect on the spinal cord (white arrows).

**Fig. 2:**
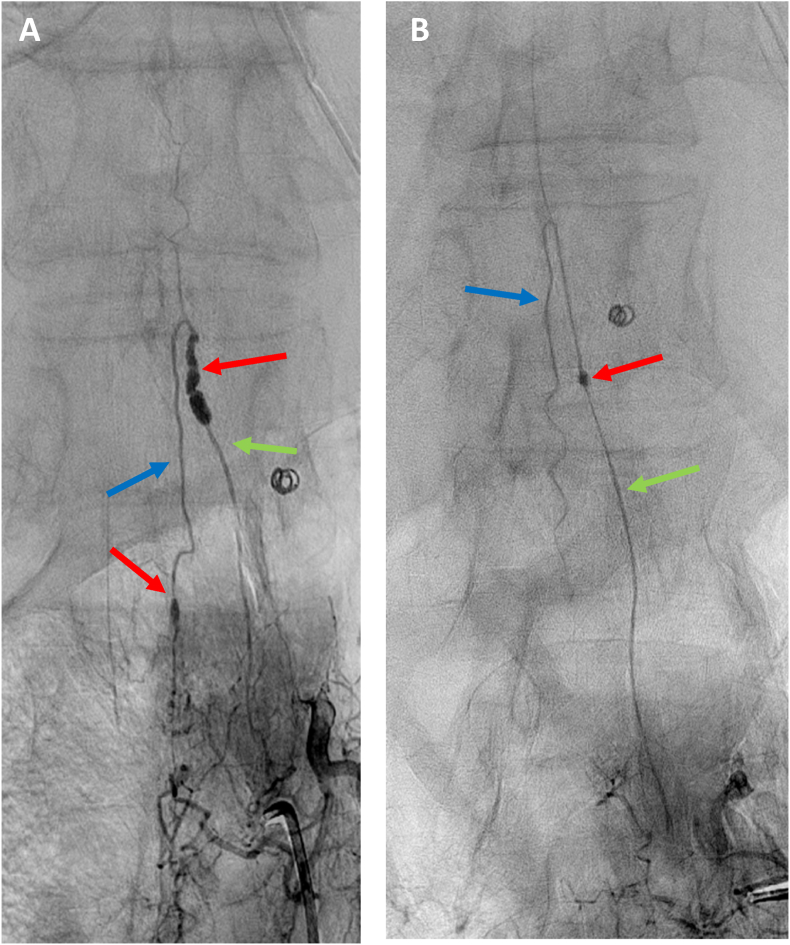
A. Selective angiogram, AP view, left L1 injection showing 2 of the fusiform spinal aneurysms (red arrows), the inflow from a radiculo-pial branch (green arrow) and the outflow represented by a posterolateral artery (blue arrow). **B**. Selective angiogram, AP view, left L2 injection showing the third fusiform aneurysm (red arrow) with its inflow represented by a radiculo-medullary branch (green arrow) and its outflow from the anterior spinal artery (blue arrow).

**Fig. 3:**
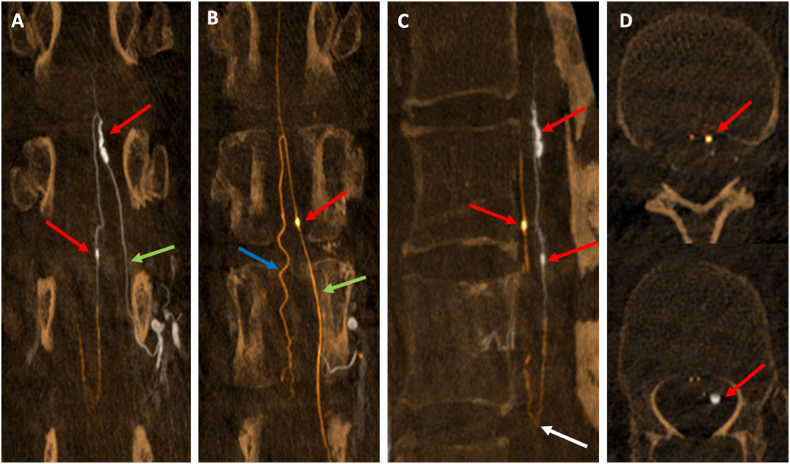
Rotational selective angiograms with Maximal Intensity Projection (MIP), fused images: Left L1 injection (background/white), Left L2 injection (overlay/orange). **A, B.** Coronal views; **A.** L1 level (white): two fusiform aneurysms (red arrows), the largest on the radiculo-pial artery (green arrow), the small one on the left posterolateral artery. **B.** L2 level (orange): fusiform aneurysm (red arrow) on the radiculo-medullary artery (green arrow); anterior spinal artery (blue arrow). **C.** Sagittal view: overview of the 3 aneurysms (red arrows) and conus anastomosis (white arrow) **D**. Aneurysms demonstrated on the axial view (red arrows).

**Fig. 4:**
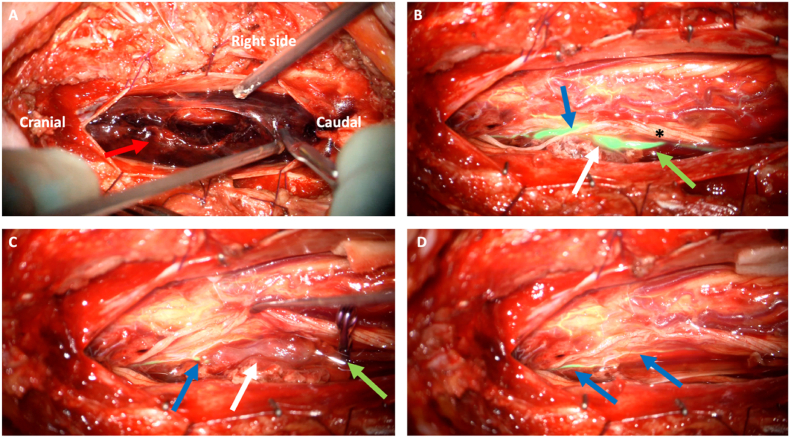
Intraoperative findings. **A**. Overview of the operative field with subdural hematoma (red arrow) compressing the spinal cord. **B**. ICG showing the angioarchitecture of the fusiform aneurysm (white arrow) with proximal inflow represented by a radiculo-pial artery (green arrow) and outflow (blue arrow) to the left posterior spinal artery. Adjacent nerve root is marked by asterisk. **C**. ICG shows maintained flow in the left posterior spinal artery during clip occlusion of the radiculo-pial artery (green arrow) with fusiform aneurysm (white arrow) and small outflow segment (blue arrow). **D.** ICG performed after excision of the aneurysm, showing preserved flow through the posterior spinal artery (blue arrows).

**Fig. 5:**
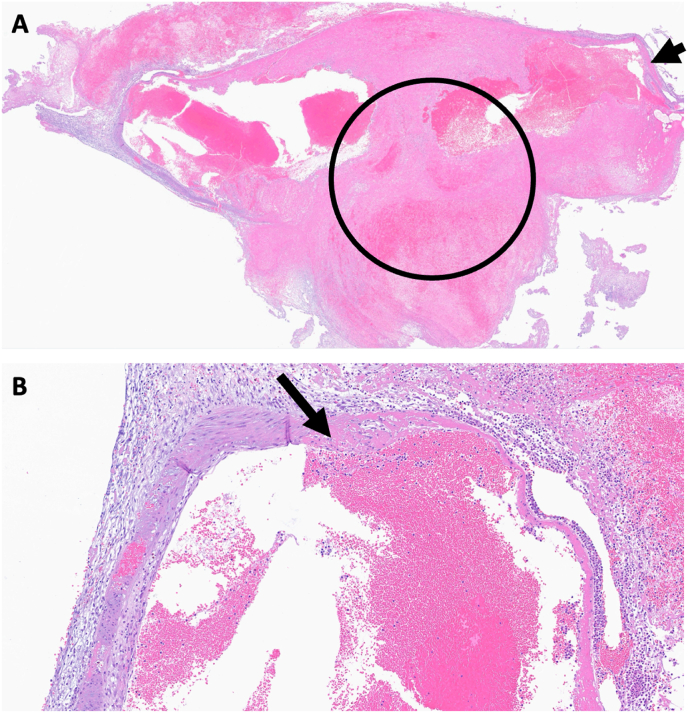
Histopathology of the excised aneurysm. **A.** Hematoxylin and eosin (HE) stained section shows artery aneurysm with endoluminal thrombosis (circle) and necrotic arterial wall (black arrow) (original magnification, ×2); **B.** Close-up view showing fibrinoid necrosis (black arrow) in the arterial wall (original magnification, ×10).

**Fig. 6:**
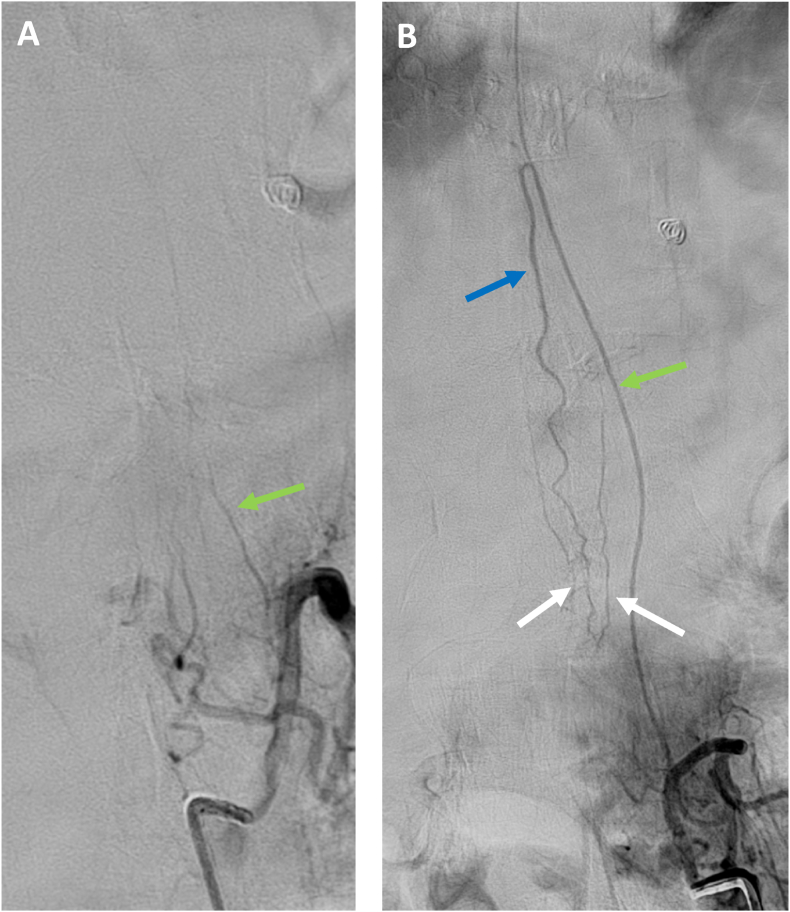
Follow up selective DSA, AP views, showing no residual (ruptured) aneurysm and spontaneous regression of the other 2 additional aneurysms previously identified. **A.** Left L1 injection showing the occluded radiculo-pial artery (green arrow) without aneurysm; **B.** Left L2 injection showing spontaneous regression of the aneurysm on the radiculo-medullary artery (green arrow); Anterior Spinal Artery (blue arrow); posterolateral arteries from anastomotic network around conus (white arrows).

A 67-year-old woman was admitted to the emergency department after experiencing acute neck pain during house cleaning. The pain radiated to her left lower limb. Initially, her symptoms improved over the following days. She subsequently experienced worsening back pain, which again radiated to her left lower limb, accompanied by sensorimotor deficits and urinary retention. On admission to hospital, an initial MRI revealed an intradural spinal hematoma with a focal perimedullary signal alteration at the T12 level, raising suspicion of an underlying vascular pathology. She was transferred to a tertiary care center, Oslo University Hospital (OUH), where a follow-up MRI and MRA of the spine showed an enlarging intradural spinal hematoma with mass effect, extending from T11 to L1 (Fig. 1). A left-sided posterior focal perimedullary signal abnormality was identified, characterized by a hypointense rim and a hyperintense core on T2-weighted imaging, with a maximum diameter of 10 mm, suggestive of a spinal aneurysm. Subsequent DSA confirmed the presence of an 11 mm segment of a (posterior) radiculo-pial branch arising from the left L1 segmental artery, with an irregular fusiform ectasia of 2 mm in maximum transverse diameter. The aneurysm was located at spinal level T12, just proximal to the radiculo-pial branch projecting into the left posterolateral spinal artery (Fig. 2, Fig. 3). The rupture of this isolated spinal aneurysm (iSA) was suspected to be the underlying cause of the spinal intradural and subarachnoid hemorrhage. Moreover, two additional fusiform, very small spinal aneurysms were found; one on the posterolateral spinal artery at the level T12/L1 (left side), and the other on an (anterior) radiculo-medullary branch at the level T12 (dominant, presumed Adamkiewicz artery, originating from the left spinal segmental artery at level L2).The patient underwent a T11-L1 laminoplasty with removal of the intradural spinal hematoma and excision of the fusiform radiculo-pial artery aneurysm, projecting into the left posterolateral spinal artery. The procedure was performed with the aid of neurophysiological intraoperative monitoring, including somatosensory evoked potentials (SSEP), motor evoked potentials (MEP), and bulbocavernosus reflex monitoring. Upon opening the dura, the hematoma drained under high pressure. The inflow vessel to the aneurysm was identified as originating from a posterior radicular artery following the left L1 nerve root, with the outflow draining into the left posterolateral spinal artery. The angioarchitecture was further characterized using indocyanine green video-angiography (ICG-VA) fluorescence imaging, guiding occlusion of the inflow vessel with an aneurysm clip (Fig. 4). Neurophysiological monitoring was conducted for 5 min, revealing no changes compared to the baseline readings. Subsequent verification with ICG-VA confirmed patency and normal flow in the posterolateral spinal artery during temporary occlusion of the radiculo-pial artery harbouring the fusiform aneurysm. The radicolo-pial artery segment with the fusiform aneurysm was than excised and sent for histological examination (Fig. 5). Surgical pathology revealed a dilated arterial wall with evidence of endoluminal thrombosis, fibrinoid necrosis, and neutrophilic granulocyte infiltration. Gram and fungal stains were also performed, ruling out infectious causes. A comprehensive rheumatologic assessment, including tests for autoantibodies such as ANA and ANCA, yielded negative results. Additionally, genomic sequencing of coding regions −20/+6 base pairs of intronic sequence in the genetic panel for thoracic aortic aneurysms and aortic dissection was performed, which also returned negative. A follow-up DSA at three months confirmed complete excision of the treated aneurysm and spontaneous regression of the two additional aneurysms (Fig. 6). The patient had recovered well with almost completely normal sensorimotor function in lower limbs and full bowel and bladder control.

## 2 Literature review

### 2.1 Methods

A comprehensive literature search was conducted to gather all reported cases of multiple iSAs, aiming to derive epidemiological, etiopathogenetic, clinical, and therapeutic insights. The PubMed database was systematically searched using MeSH terms and targeted keywords to identify relevant studies. Additionally, extensive cross-referencing was conducted by screening case reports, case series, and review articles for descriptions of multiple iSAs. All available data on multiple iSAs were extracted for analysis. Our search strategy yielded 10 relevant articles published between 1950 and 2024, reporting cases of multiple iSAs. The extracted data encompassed demographic and clinical data, aneurysm morphology and topography, detection methods, significant associated comorbidities, pathology findings (when available), and follow-up outcomes. A summary of these data is presented in Table 1.

**Table 1:** Summary of cases reported with multiple-iSAs in the literature.

Authors	Year	No. multiple iSA	Location	Sex	Age	Ruptured	Clinical onset	Treatment	Outcome (mRS)^a^	Follow-up
Yoong et al.	1993	3	cervical	F	55	Yes	acute	conservative	6	5 days
Massand et al.	2005	2	lumbar	M	69	Yes	acute	surgery (resection)	3	NA
Longatti et al.	2008	2	thoraco-lumbar	F	54	Yes	subacute	conservative	0–2^b^	6 months
Sato et al.	2012	2	thoracic	F	67	Yes	acute	conservative	3	18 months
Nagahata et al.	2013	2	cervical	F	70	Yes	acute	endovascular (coiling)	4	NA
Ronchetti et al.	2014	2	thoracic	F	51	Yes	acute	surgery (resection)	0–2^b^	NA
Renieri et al.	2018	3	thoracic	NA	NA	Yes	NA	surgery (trapping)	0–3^c^	8 months
Renieri et al.	2018	2	NA	NA	NA	Yes	NA	surgery (resection)	0–3^c^	1 month
Halabi et al.	2021	4	thoracic	F	35	Yes	subacute	surgery (clipping and resection)	2–4^d^	24 months
McGuire et al.	2023	3	thoraco-lumbar	F	60	Yes	acute	conservative	1–3^e^	1.5 months
McGuire et al.	2023	2	thoraco-lumbar	M	64	Yes	acute	conservative	1–3^e^	6 months
Zhou et al.	2024	4	cervical	NA	16	Yes	acute	endovascular (coiling)	6	5 months
Our case	2024	3	thoraco-lumbar	F	67	Yes	subacute	surgery (clipping and resection)	2	14 months

a Modified Ranking Scale (mRS) scoring system was retrospectively applied if not primarily reported by the original article, based on description of clinical examinations to enable comparability. NA = not available.

b Information enables scoring of mRS between 0 and 2.

c Only “good outcome” was reported, therefore mRS will be in the range of 0–3.

d information enables scoring of mRS between 2 and 4.

e Information enables scoring of mRS between 1 and 3.

## 3 Results

### 3.1 Epidemiology

Including our case, data from 13 patients with a total of 34 iSAs were included for analysis. Due to the extreme rarity of this condition, only case reports were available, and the true incidence of multiple iSAs remains unknown. Most reported cases involved adult females. Age information was available for 11 of the 13 patients, with an average of 55.3 years of age (range 16–70). Sex data was available for 10 patients, of whom 8 were female and 2 males. Despite the small sample size, there was a noticeable predominance of middle-aged women in the reported cases.

### 3.2 Location, morphology and multiplicity

Our analysis of multiple iSAs revealed an average of 2.6 aneurysms per patient. In contrast to intracranial aneurysms, which are predominantly saccular, the majority of iSAs were fusiform in shape. Specifically, 10 of 13 patients (77%) had fusiform aneurysms, 2 patients (15.4%) had dissecting aneurysms and 1 patient (7.6%) had a saccular aneurysm. Most of these aneurysms (61.7%) were located in the thoracolumbar region, followed by the cervical (26.5%) and lumbar (11.8%) regions. Artery of aneurysm origin was described as: 11 aneurysms (32.4%) on radiculo-medullary arteries, 7 (20.6%) on the anterior spinal artery, 6 (17.6%) on radiculo-pial arteries, and 5 each (14.7%) on radicular arteries and the posterior spinal artery (Table 2).

**Table 2:** Summary of relevant features of patients harboring multiple-iSAs.

Patient No.	Location	Vessel of origin	Type of aneurysm	Associated condition	Spontaneous regression of non-treated iSAs
1	Cervical	Radicular artery	Fusiform	Lymphoma	No
2	Lumbar	Radicular artery	Dissecting	–	No
3	Thoraco-lumbar	Anterior spinal artery	Fusiform	Multiple visceral aneurysms	Yes
4	Thoracic	Radiculo-pial artery	Fusiform	Pearl and string pattern	Yes
5	Cervical	Radiculo-medullary artery	Saccular	Bilateral carotid and vertebral rete mirabile	NA
6	Thoracic	Posterior spinal artery	Fusiform	–	Yes
7	Thoracic	Radiculo-medullary and radiculo-pial arteries	Fusiform	–	NA
8	NA	Radiculo-medullary and radiculopial arteries	Fusiform	–	NA
9	Thoracic	Posterior spinal artery, Anterior spinal artery	Fusiform	Polyarteritis nodosa, multiple visceral aneurysms	NA
10	Thoraco-lumbar	Radiculo-medullary arteries	Fusiform	–	Yes
11	Thoraco-lumbar	Radiculo-medullary	Dissecting	–	Yes
12	Cervical	Posterior spinal artery	Fusiform	Suspected connective tissue disease	NA
13	Thoraco-lumbar	Radiculo-pial artery	Fusiform	–	Yes

NA = not available.

### 3.3 Possible triggers for the development of multiple iSAs

The potential etiological factors contributing to the development of multiple iSAs were analyzed. In 3 cases, the presence of multiple intra-visceral aneurysms was reported, while in another three cases there was a confirmed or suspected immune-mediated pathology. Additionally, in two cases, including ours, angiographic examination revealed a “pearls and strings” pattern affecting medium and small caliber arteries, suggesting the presence of immunoinflammatory disease as a contributing factor, likely resulting in the weakening of the vascular wall.

### 3.4 Histological features

The literature provided histological information on multiple spinal aneurysms in three cases involving rupture. The first case was a fusiform aneurysm of a cervical radicular artery in a patient with a non-Hodgkin's lymphoma, where histology showed a picture of necrotizing vasculitis, fibrinoid necrosis and vessel wall infiltration with lymphocytes and polymorphonuclear cells (Yoong et al., 1993). The second case involved a ruptured spinal aneurysm at level T7 in a patient with polyarteritis nodosa (Halabi et al., 2021). Histological examination of the vessel wall revealed fibrinoid necrosis and infiltration of inflammatory cells, consistent with a vasculitic pattern.

In the third case, histopathology confirmed the dissecting nature of the aneurysm. As a result, much of the histological information regarding multiple spinal aneurysms must be extrapolated from studies focusing on single spinal aneurysms (Massand et al., 2005). Surgically excised iSAs exhibit a thinned vascular wall with a disrupted elastic lamina, organized thrombi and granulation tissue. In some cases a dissection of the vessel with an intramural hematoma and a segmental dilatation of the parent artery has been reported (Massand et al., 2005). Ruptured spinal artery aneurysms appear to have a tendency for spontaneous thrombosis (Ikeda et al., 2016). In several cases, the presence of organized blood clots without discernible intima or endothelial lining suggested a pseudoaneurysmal nature (Geibprasert et al., 2010).

### 3.5 Diagnosis and clinical presentation

All patients with multiple iSAs in the gathered studies had a clinical presentation following aneurysm rupture. Selective spinal digital subtraction angiography (DSA) is considered the gold standard to confirm diagnosis and to enable the detailed assessment of multiple aneurysms. Patients presented with acute onset of symptoms triggering hospital admission and further investigations. Most commonly, patients presented with sudden onset of: 1) new neurological deficit (such as lower extremity weakness and/or paresthesia); 2) back pain; 3) headache and nuchal rigidity. The presence of nuchal rigidity, as a clinical surrogate of subarachnoid hemorrhage, was not confined to cervical or cranio-cervical location, but also reported in caudal aneurysm localizations (McGuire et al., 2023).

### 3.6 Treatment

Conservative aneurysm treatment was reported in 5 out of 13 cases (38.5%) (McGuire et al., 2023; Ronchetti et al., 2015). Surgical treatment was performed in 6 cases (46.1%) including 5 aneurysm resections and 1 trapping [9,17,2324]. Endovascular treatment (coil embolization) was performed in 2 cases (15.4%) (Nagahata et al., 2013; Zhou et al., 2024).

### 3.7 Outcomes and follow-up

Outcomes were reported according to the modified Rankin Scale (mRS) assessed at the time of the last follow-up. For comparability reasons, we retrospectively applied mRS scores for cases where the clinical follow-up information enabled estimation of mRS and mRS was not specifically reported. A favorable neurological outcome (defined as an mRS score from 0 to 3) was observed in 9 out of 13 cases (69%). The available information indicated that an mRS score between 2 and 4 could be determined for 1 out of the 13 patients. One patient had a poor outcome with moderate disability and mRS score of 4. Two patients died during follow-up, one due to subarachnoid hemorrhage 5 months after treatment of one of the multiple aneurysms by coiling and the other due to systemic vasculitis and underlying MALT-lymphoma. It is noteworthy that there is significant heterogeneity in the evaluation of outcome and length of reported follow-up between studies (Table 1), hampering a more formal analysis of outcome and evaluation of long-term treatment result.

## 4 Discussion

Multiple isolated spinal aneurysms are exceedingly rare lesions. All reported cases in the literature were identified following rupture, leading to subarachnoid hemorrhage and subsequent clinical symptoms. Including our case, we have identified 13 patients with a total of 34 aneurysms. The majority of aneurysms was reported in middle-aged women. Aneurysms predominantly involved a radiculo-medullary artery, particularly along the ascending branch of the inflow vessel, and tended to cluster within the same anatomical (metameric) region. In some cases, they were associated with vasculitis affecting multiple vascular territories (McGuire et al., 2023).

Based on these observations, two distinct patterns of multiple iSAs can be considered: one driven by hemodynamic factors and the other by an inflammatory insult compromising the vascular wall integrity.

### 4.1 Epidemiology and etiology of multiple iSAs

Little is known about the epidemiology of multiple iSAs. They occur in all age groups and in both sexes, but seem to have a greater predisposition for middle-aged women (McGuire et al., 2023). They most commonly occur in the thoracic spine, presumably just due to the greater length of that medullary segment. Several predisposing conditions for multiple iSAs have been described: Aortic coarctation, bilateral stenosis of vertebral arteries and connective tissues disease are among the most associated conditions(Ashour et al., 2015). Differently from their intracranial counterpart, iSAs are more commonly fusiform shaped and pseudoaneurysms as a result of a dissection of the vessel wall (Geibprasert et al., 2010). Often a well-defined aneurysm neck is not present, and they tend to occur along arterial segments not associated with branching points. Moreover, they frequently exhibit features of endoluminal thrombosis with potential for distal spreading of emboli (Tenorio et al., 2021; Ikeda et al., 2016). Of note, cases described in the literature were discovered following the rupture of one of the aneurysms, perhaps highlighting a greater propensity to rupture for multiple spinal aneurysms.

### 4.2 The concept of metameric contiguity in multiple iSAs

Embryologically, the segmental vessels supplying the spinal cord originate from a bilateral network along the anterolateral surface of the medulla, connecting segmental feeders at the cervical, thoracic, and upper lumbar levels to the aorta. This network undergoes regression, eventually forming the anterior spinal artery as a single anterior longitudinal system, supplied by radiculo-medullary branches from persisting segments following the ventral nerve roots, together with the posterolateral network of arteries, supplied by radiculo-pial branches following the dorsal roots(Thron et al., 2022). The segmental feeders, particularly at the lumbar level, regress significantly by the fourth month of embryonic development, finalizing the arterial supply distribution to the spinal cord. This involution, most pronounced in the caudal segments, may explain the higher incidence of spinal aneurysms in thoracic radiculo-medullary vessels, as these are more numerous. Multiple iSAs appear to cluster in contiguous metameric segments. This pattern suggests that anomalies in the regression process may predispose to disease development at contiguous levels, as frequently observed in multiple iSAs.

### 4.3 Hemodynamic hypothesis for multiple iSAs

Multiple iSAs tend to occur in metameric regions that are contiguous. This suggests that locoregional hemodynamic alterations may promote the formation of flow-related multiple iSAs. The literature shows an inverse relationship between the number and caliber of anterior radiculo-medullary arteries supplying the spinal cord: fewer arteries (2–5) result in larger vessels with a so-called paucisegmental pattern, while more arteries lead to smaller vessels with a plurisegmental pattern(Schneider and Ule, 1980). A reduced vascular supply may increase hemodynamic resistance locally, contributing to the development of multiple aneurysms within a single metameric district. This theory could be supported by the observation that congenital conditions like aortic coarctation can cause the anterior spinal artery to act as a collateral vessel, leading to increased flow and aneurysm formation. These factors together suggest that regional hemodynamic conditions may predispose to the formation of flow-related aneurysms.

### 4.4 Immuno-inflammatory diseases and multiple iSAs

The development of isolated spinal aneurysms (iSAs) is linked to hemodynamic stress and vascular wall damage, often associated with connective tissue disorders or autoimmune conditions. The observed correlation between some multiple spinal aneurysms and conditions such as bilateral carotid rete mirabile or vertebral stenosis highlights the potential role of hemodynamic factors in the pathogenesis of multiple iSAs (Nagahata et al., 2013). In addition, concomitant presence of multiple iSAs and visceral aneurysms (i.e. hepatic arteries, renal arteries) with a pearl and strings angiographic appearance, present in connective tissue diseases has been described (Renieri et al., 2018). Therefore, ongoing inflammatory processes can sustain damage and weakening off the vessel from which aneurysmal dilations can result. This can lead to what we call wall-weakening aneurysms. Connective tissue disease should be suspected in case of multiple fusiform aneurysms. A further area under investigated in the reported literature is the role of tissue biopsy, even in superficial and not directly-involved vascular territories, which could be useful for revealing immune-mediated vascular diseases that are difficult to diagnose (Halabi et al., 2021). In case of immune-mediated conditions, medical treatment of the underlying disease is likely to be the best therapy.

### 4.5 Clinical presentations

Multiple iSAs typically present with rupture, resulting in subarachnoid hemorrhage(Alomari et al., 2022; Abdalkader et al., 2021). The onset of a new neurological deficit, followed by a sudden onset of back pain are the most frequent symptoms. In most cases, pain location corresponds to the site of the ruptured aneurysm, although it may later migrate. Noteworthy, ruptured iSAs may present with intracranial symptoms (Cavuşoğlu et al., 2010; Horio et al., 2015; Sung et al., 2015; Yokosuka et al., 2020; Chen et al., 2022). These may include headache, nausea, photophobia, and neck stiffness(Bergeron et al., 2021; Ray et al., 2013). In these cases, a high level of suspicion is needed for considering a ruptured spinal artery aneurysm as a cause of a posterior cranial fossa-SAH with negative cerebral angiography or perimesencephalic SAH (Abdalkader et al., 2021; Malhotra et al., 2021). Spinal cord compression in case of mass effect from a spinal hematoma including the possibility of quadriplegia (Handa et al., 1992; Santana-Ramírez et al., 2013) or cauda equina syndrome are further possible presentations of ruptured iSAs.

### 4.6 Diagnosis

Spinal CT and CT angiography and/or spinal MR including contrast enhanced MR angiography are the initial studies recommended for patients with spinal subarachnoid hemorrhage. However, these tests can sometimes be negative due to the small size of the aneurysm or its obscuration by a spinal hematoma(Marovic et al., 2013). Therefore, conventional angiography with selective segmental contrast injections remains the gold standard for diagnosis and treatment planning. It is also indicated to perform spinal angiography in cases of subarachnoid hemorrhage in the posterior cranial fossa with negative cerebral angiography, as well as in perimesencephalic SAH cases when spinal pathology is suspected (Abdalkader et al., 2021; Malhotra et al., 2021). Given the potential for spontaneous aneurysm resolution through endoluminal thrombosis, repeat imaging may be advisable just before finalizing the treatment plan.

### 4.7 Treatments

There are no standardized treatment algorithms for multiple iSAs, with management options including conservative, surgical, or endovascular interventions(Tanweer et al., 2012). Multiple iSAs are typically discovered only after the rupture of one aneurysm, necessitating that treatment initially focuses on the ruptured aneurysm, followed by evaluation and management of the others. Given the potential for spontaneous resolution of a significant proportion of iSAs, conservative management should be carefully considered for non-ruptured aneurysms when selecting the overall treatment strategy. Given the lack of systematic treatment guidelines, the establishment of multicenter registries should be encouraged to track and analyze rare cases like these. Such registries would not only improve data collection, but also facilitate collaborations across institutions, ultimately contributing to the development of evidence-based treatment strategies and a more comprehensive understanding of treatment nuances for multiple iSAs.

### 4.8 Surgery

Surgical treatment is particularly indicated when the aneurysm or associated hemorrhage exerts significant mass effect causing neurological deficits. Surgical techniques vary, including clipping, trapping with aneurysm resection, or wrapping (Ren et al., 2018; Vishteh et al., 1997). Posterior approaches to the spinal cord are the primary surgical route for accessing spinal aneurysms(Priola et al., 2019; Aljuboori et al., 2018; Scullen et al., 2023). Given the predominantly fusiform nature of these lesions, clipping or trapping often requires sacrificing the inflow artery, which is feasible only if the aneurysm is fed by a radicular vessel with sufficient collaterals or a small expendable radiculo-pial branch, without leading to medullary ischemia (Longatti et al., 2008). We think the use of intraoperative Indocyanine green angiography (ICG) and neurophysiological monitoring (IONM) are crucial in guiding the surgical strategy of vessel sacrifice.

### 4.9 Endovascular

Endovascular treatment of spinal aneurysms presents several challenges. Selective coiling without parent vessel occlusion is viable only for saccular side-wall aneurysms with a well-defined, small neck. However, most spinal aneurysms lack a true neck or are fusiform in shape, which increases the risk of spinal infarction during embolization. An endovascular treatment would typically imply liquid embolic material (glue) and vessel sacrifice, with the risk of distal migration of embolic material to the medulla, and with technical challenging catheterization of the very small, often tortuous radicular branches. Also, test occlusion with ongoing IONM has not been an established tool in iSA treatment by endovascular means, decreasing the safety of this approach. This is clearly reflected in the paucity of reports in endovascular treatment for iSAs. Considering this, only few of the reported iSA cases were treated by endovascular techniques.

### 4.10 Conservative

Spontaneous resolution of iSAs has been documented in several cases, suggesting that conservative management may be appropriate, particularly in patients without the need for spinal canal decompression or when aneurysms are not amenable to straightforward treatment (e.g. anterior spinal artery aneurysms). Although the limited number of reported cases prevents robust statistical analysis, existing clinical data indicate that iSAs are prone to thrombosis, with a notable proportion being pseudoaneurysms resulting from vessel wall dissection (Ikeda et al., 2016). These pseudoaneurysms can regress spontaneously over time through endoluminal thrombus formation, a process that supports a conservative approach in more fragile patients. However, the risk of re-rupture in iSAs remains unclear due to inconsistencies in long-term follow-up. Currently, there are no objective predictors of spontaneous regression or the likelihood of re-hemorrhage. Future research efforts should focus on identifying clinical and radiological parameters, including systematic follow-up of pooled cases that could predict the natural history of iSAs and guide treatment decisions.

## 5 Conclusion

This study highlights the challenges of diagnosing and managing the rare condition of multiple isolated spinal aneurysms. Through a review of 13 cases, we found that iSAs often affect middle-aged women, are predominantly fusiform, and cluster in contiguous metameric regions, likely due to hemodynamic or immune-mediated factors.

Management should be tailored, with ruptured aneurysms requiring prompt intervention and non-ruptured aneurysms often managed conservatively due to their potential for spontaneous resolution. Tools like ICG-VA and IONM enhance surgical safety by reducing risks. The rarity of iSAs and the lack of standardized guidelines call for collaborative research and multicenter registries to improve treatment strategies and outcomes.

## Ethical statement

The patient gave written informed consent for this case presentation.

## Declaration of generative AI and AI-assisted technologies in the writing process

During the preparation of this work, the author(s) used ChatGPT, an AI-assisted writing tool, to assist in refining the text for clarity, conciseness, and grammar. After using this tool, the author(s) reviewed and edited the content as needed and take full responsibility for the content of the publication.

## Declaration of competing interest

The authors declare that they have no known competing financial interests or personal relationships that could have appeared to influence the work reported in this paper.
